# The role and potential of digital breast tomosynthesis in neoadjuvant systemic therapy evaluation for optimising breast cancer management: a pictorial essay

**DOI:** 10.1093/bjr/tqae252

**Published:** 2024-12-26

**Authors:** Luciano Mariano, Luca Nicosia, Antuono Latronico, Anna Carla Bozzini, Valeria Dominelli, Davide Pupo, Filippo Pesapane, Maria Pizzamiglio, Enrico Cassano

**Affiliations:** Division of Breast Radiology, Department of Medical Imaging and Radiation Sciences, IEO European Institute of Oncology, IRCCS, 20141, Via Ripamonti 435, Milano, Italy; Division of Breast Radiology, Department of Medical Imaging and Radiation Sciences, IEO European Institute of Oncology, IRCCS, 20141, Via Ripamonti 435, Milano, Italy; Division of Breast Radiology, Department of Medical Imaging and Radiation Sciences, IEO European Institute of Oncology, IRCCS, 20141, Via Ripamonti 435, Milano, Italy; Division of Breast Radiology, Department of Medical Imaging and Radiation Sciences, IEO European Institute of Oncology, IRCCS, 20141, Via Ripamonti 435, Milano, Italy; Division of Breast Radiology, Department of Medical Imaging and Radiation Sciences, IEO European Institute of Oncology, IRCCS, 20141, Via Ripamonti 435, Milano, Italy; Division of Breast Radiology, Department of Medical Imaging and Radiation Sciences, IEO European Institute of Oncology, IRCCS, 20141, Via Ripamonti 435, Milano, Italy; Division of Breast Radiology, Department of Medical Imaging and Radiation Sciences, IEO European Institute of Oncology, IRCCS, 20141, Via Ripamonti 435, Milano, Italy; Division of Breast Radiology, Department of Medical Imaging and Radiation Sciences, IEO European Institute of Oncology, IRCCS, 20141, Via Ripamonti 435, Milano, Italy; Division of Breast Radiology, Department of Medical Imaging and Radiation Sciences, IEO European Institute of Oncology, IRCCS, 20141, Via Ripamonti 435, Milano, Italy

**Keywords:** breast cancer, tomosynthesis, neoadjuvant therapy

## Abstract

Neoadjuvant therapy (NT) has become the gold standard for treating locally advanced breast cancer (BC). The assessment of pathological response (pR) post-NT plays a crucial role in predicting long-term survival, with contrast-enhanced MRI currently recognised as the preferred imaging modality for its evaluation. Traditional imaging techniques, such as digital mammography (DM) and ultrasonography (US), encounter difficulties in post-NT assessments due to breast density, lesion changes, fibrosis, and molecular patterns. Digital breast tomosynthesis (DBT) offers solutions to prevalent challenges in DM, such as tissue overlap, and facilitates a comprehensive assessment of lesion morphology, dimensions, and margins. Studies suggest that DBT correlates more accurately with pathology than DM and US, showcasing its potential advantages. This pictorial essay demonstrates the potential of DBT as a complementary tool to DM for assessing pR after NT, including instances of true- and false-positive assessments correlated with histopathological findings. In conclusion, DBT emerges as a valuable adjunct to DM, effectively addressing its limitations in post-NT assessment. The technology's potential to diminish tissue overlap, improve discrimination, and provide multi-dimensional perspectives demonstrates promising results, indicating its utility in scenarios where MRI is contraindicated or inaccessible.

## Introduction

Neoadjuvant therapy (NT) denotes the systemic treatment of breast cancer (BC) before potentially radical surgery aimed to reduce the burden of primary malignancy, allowing less extensive procedure with favourable aesthetic outcomes, and also to provide a prognostic and predictive assessment of response to systemic therapies to guide adjuvant treatment recommendations.^1^

Initially conceived exclusively as preoperative chemotherapy, there has been growing interest in expanding the role of endocrine and targeted therapy for neoadjuvant purposes as the standard of care for patients with locally advanced (stage IIB-IIIC) BC^1^^,^^2^ as well as for operable tumours (stages I-IIA), when conservative surgery is not immediately practicable (eg, high tumour-to-breast ratio or specific patient's breast anatomy).^3^^,^^4^ Moreover, achieving a complete pathological response (pCR) after NT, defined as the absence of residual invasive disease in the breast parenchyma and/or lymph node (ypT0ypN0),^5^ is a predictive indicator of successful long-term survival in BC patients.^6^^,^^7^ This is particularly notable in tumour subtypes with higher biological aggressiveness, like HER2-positive and triple-negative cancers,^5^ where NT is always recommended.

Accurate clinical and radiological assessment during and after NT is crucial for possible treatment adjustments and further surgical management.^8^ This evaluation, involving physical examination and various imaging modalities such as digital mammography (DM), ultrasonography (US), contrast-enhanced mammography (CEM), and contrast-enhanced MRI, aims to confirm pathology and document disease extent, with MRI currently considered the imaging technique of choice.^9^

In this pictorial essay, we aim to illustrate the possible advantages or limitations of digital breast tomosynthesis (DBT) as an additional tool to conventional DM in evaluating the pathological response (pR) after NT: we provide an educational review with illustrative images and descriptive cases of true-positive and false-positive assessment of the residual disease following NT attributed to DBT with histopathological correlation.

## NT and imaging evaluation

According to the NCCN recommendations, a multidisciplinary team should reach a consensus on which studies should be performed in patients undergoing NT.^10^ Contrast-enhanced imaging techniques, particularly MRI, are currently the most sensitive tool for assessing disease response during and after NT. MRI has been shown to offer a more accurate estimate than conventional radiological techniques, providing relevant information on tumour extension, dense breast tissue on DM, internal mammary adenopathy or suspected pectoralis muscle and chest wall invasion.^11^^,^^12^ In recent years, CEM fusing the DM morphological data with functional information using an intravenously injected iodinate contrast agent has found significant application in this context. A recent meta-analysis demonstrated an excellent accuracy of CEM in detecting residual disease after NT, with sensitivity and specificity rates of 80.7% and 94%, respectively.^13^ In addition, prospective studies comparing CEM and MRI showed a superior positive predictive value in assessing response to NT; also, in complete response evaluation, CEM showed higher sensitivity and specificity (100% and 84%) than MRI (87% and 60%), and a higher concordance with invasive tumour size (0.70 vs 0.66, respectively).^14^^,^^15^ However, high costs and the uneven territorial distribution of equipment may limit the widespread large-scale adoption of these techniques.

As already reported in the literature, DM and US are commonly used tools for tumour size evaluation at diagnosis time^16^^,^^17^; nevertheless, changes resulting from NT can increase the complexity of this assessment. Although DM shows statistically lower accuracy than the US, it also tends to demonstrate a lower overestimation rate. In addition, DM only measures residual tumour in half of the cases, a significantly lower rate compared to US.^17^ Several aspects may influence the accuracy of DM in the assessment of tumour response after NT^8^: (1) breast composition and density; (2) lesion change density; (3) development of fibrotic areas; (4) DM tumour characteristics; (5) the presence of microcalcifications; (6) fragmentation; (7) molecular profiles and response patterns.^17–20^

In this scenario, DBT could offer an additional tool to overcome DM limitations. It is a pseudo-3D evolution of conventional DM technology that uses traditional X-rays and a digital detector to create cross-sectional breast tissue images (or slices).^21^ Due to these technical aspects, DBT can reduce tissue overlap and the masking effect of breast density, facilitating discrimination between typical tissue structures and lesions and enabling better assessment of the lesion morphology, size, and margins.^22^^,^^23^ Evidence from the literature supports the heightened sensitivity of DBT compared to DM in detecting lesions in both fatty and dense breasts.^24–26^ Moreover, several authors concur on the enhanced correlation between tumour sizes measured by DBT and confirmed by definitive histology, exceeding the capabilities of DM. Finally, DBT could also contribute to overcoming some of the limitations induced by NT in the assessment of response by DM alone, primarily related to necrosis, fragmentation, and fibrosis.^22^^,^^23^^,^^27^^,^^28^

To date, limited studies have examined the accuracy of DBT in assessing response after NT. Park et al reported a better correlation between DBT and MRI with the pathological evaluation of residual disease than DM and US.^29^ Moreover, in patients with intralesional metal clips implanted for NT monitoring, DBT may be a viable alternative to MRI due to its better clip visualisation by exploiting the metal’s sensitivity to X-rays^30^ and its lower susceptibility to artefacts from some localization devices that may limit accuracy in the residual disease assessment on MRI.^31^^,^^32^

These results align with previous studies,^33^^,^^34^ proposing the addition of DBT at DM as an alternative imaging approach to the functional technique in assessing patients undergoing NT. Improving the diagnostic accuracy of DM and facilitating the correlation between macroscopic disease assessment and pathological evaluation, this integration could provide a cost-effective and widely available tool, enhancing the care of BC patients undergoing NT without necessitating contrast-enhanced techniques.

Automated breast ultrasound (ABUS), a computer-assisted technique for assessing the whole breast using an automated linear 6-14 MHz transducer selected according to breast thickness, is also emerging as a promising tool. It could complement DBT-DM assessment in terms of both efficacy and cost reduction.

Hatzipanagiotou et al found no statistical difference between US and ABUS (*P* = .47) in predicting pathological tumour size.^35^ Additionally, ABUS has shown a good correlation with MRI in evaluating tumour response, suggesting its potential utility post-NT, although the study sample size was small.^36^^,^^37^ Peng et al, analysing 156 patients to compare ABUS with other imaging techniques in the assessing accuracy of residual tumour size, demonstrated a moderate ABUS correlation with pathologic size of residual invasive tumours but better accuracy in residual tumour size measurement than other imaging methods (*P* < .05).^38^ These findings suggest that ABUS could be a valuable tool for post-NT assessment of BC, offering benefits in investigation time, reproducibility across different investigators, and better preoperative planning.^35^ Indeed, its 3D recording technique provides additional preoperative information about tumour extension and tumour-to-breast volume ratio, aiding the surgical approach. However, ABUS may not detect peripherally localized lesions, particularly in large breasts. Whole-breast scanning with additional acquisitions from the upper and lower breast regions may address this limitation.^39^

Nevertheless, some challenges and limitations must be addressed. Although radiation exposure in these patients is not a major concern due to their undergoing post-surgery radiotherapy protocols, it should be remembered that DBT utilizes 2D synthetic images^40^ with comparable (or slightly higher) radiation dose values than conventional 2D imaging but still below the limit set by the European Guidelines for Screening Mammography.^41^ In addition, a careful axillary evaluation by the US is always necessary. US sensitivity and specificity for diagnosis and assessing residual disease in the regional nodes of up to 94% and 98%, respectively, were demonstrated.^42^ Data from the ACOSOG Z071 showed that lymph node size, the cortical thickness of the most abnormal lymph node, and the presence or loss of the fatty hilum are the most important criteria to evaluate residual nodal disease status after NT.^43^

## Role of DBT in improving DM accuracy

We portray the spectrum of the major factors that make determining tumour extent by DM difficult following NT and the possible advantages of DBT use.

### Breast composition and density

**Figure 1.**
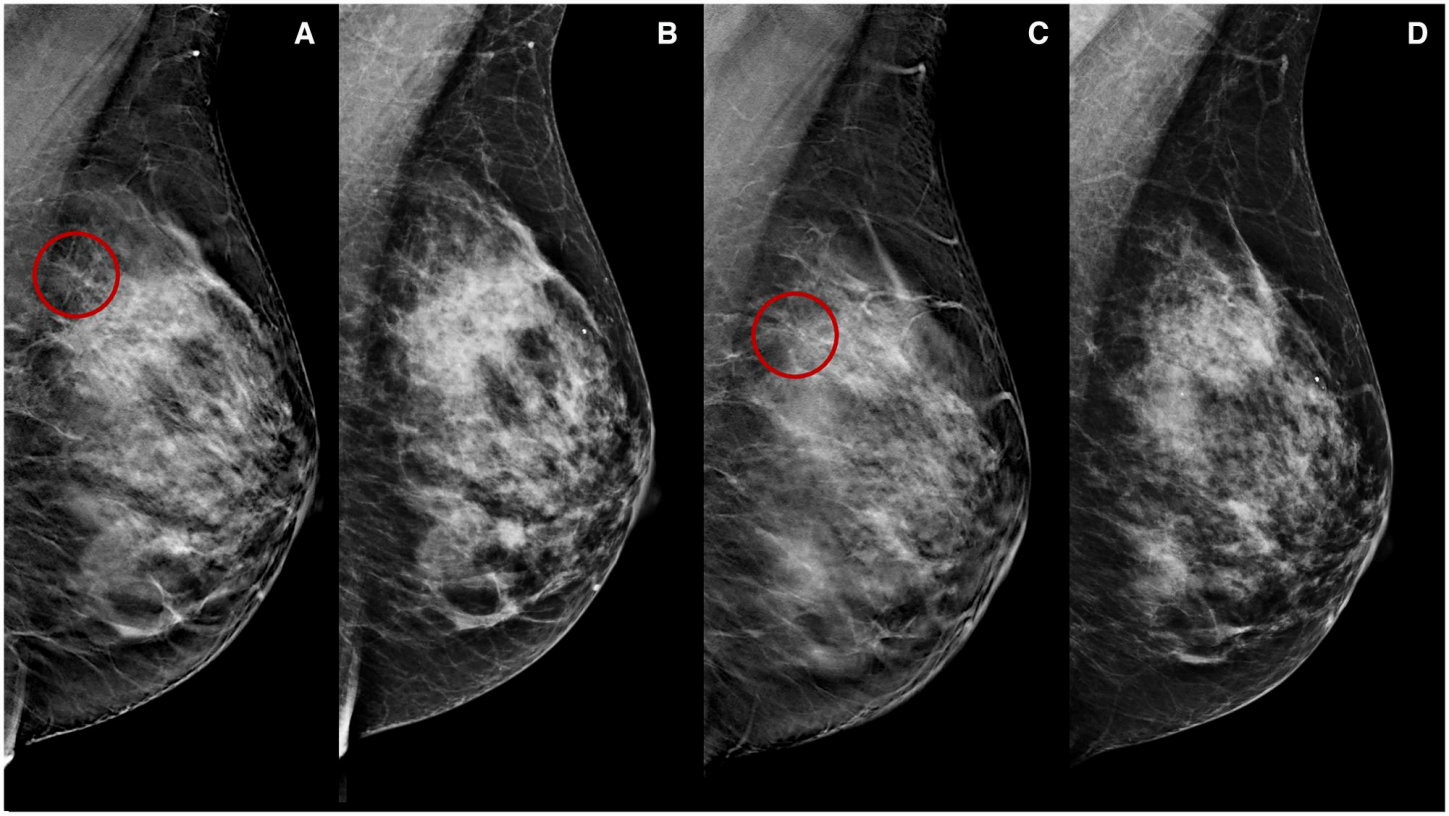
A 46-year-old woman underwent NT (four cycles of epirubicin-cyclophosphamide followed by weekly paclitaxel for 12 weeks) for an invasive ductal carcinoma cT1cN0, G3 (ER 0%, PgR 0%, Ki-67 65%, HER2 1+). Medio-lateral-oblique DBT before (A) and following (C) NT show a parenchymal distortion in the upper half of the left breast (rims) obscured by high fibroglandular tissue density on DM (B, D).

Increased radiopacity in breast composition (categorised as BI-RADS C and D^44^) is associated with a low DM sensitivity in tumour lesion detection, considering that most mass-forming BCs have an equal or higher density than a similar volume of normal fibroglandular tissue.^45^ By reducing the masking effect of breast density, DBT contributes to better detecting and identifying the tumour or residual lesion after NT (Figure 1).

### Lesion change density

**Figure 2.**
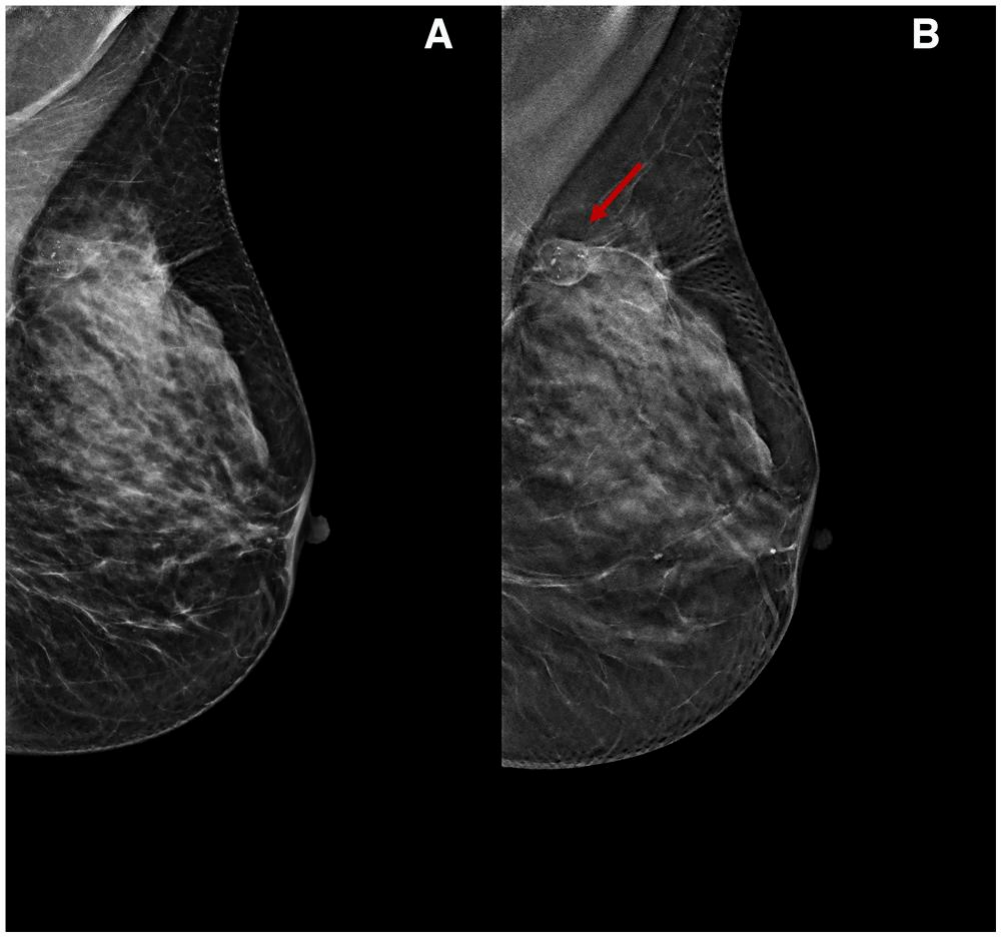
A 55-year-old woman underwent NT (four cycles of epirubicin-cyclophosphamide followed by weekly paclitaxel for 12 weeks) for an invasive ductal carcinoma cT2cN0, G2 (ER 10%, PgR 0%, Ki-67 40%, HER2 0). Medio-lateral-oblique DM (A) and DBT (B) following NT. There is a lucent oval area with a radiopaque rim and some microcalcifications inside in the upper quadrants of the left breast (arrow), in keeping with liponecrotic phenomena which is seen on DBT but not clearly seen on DM.

**Figure 3.**
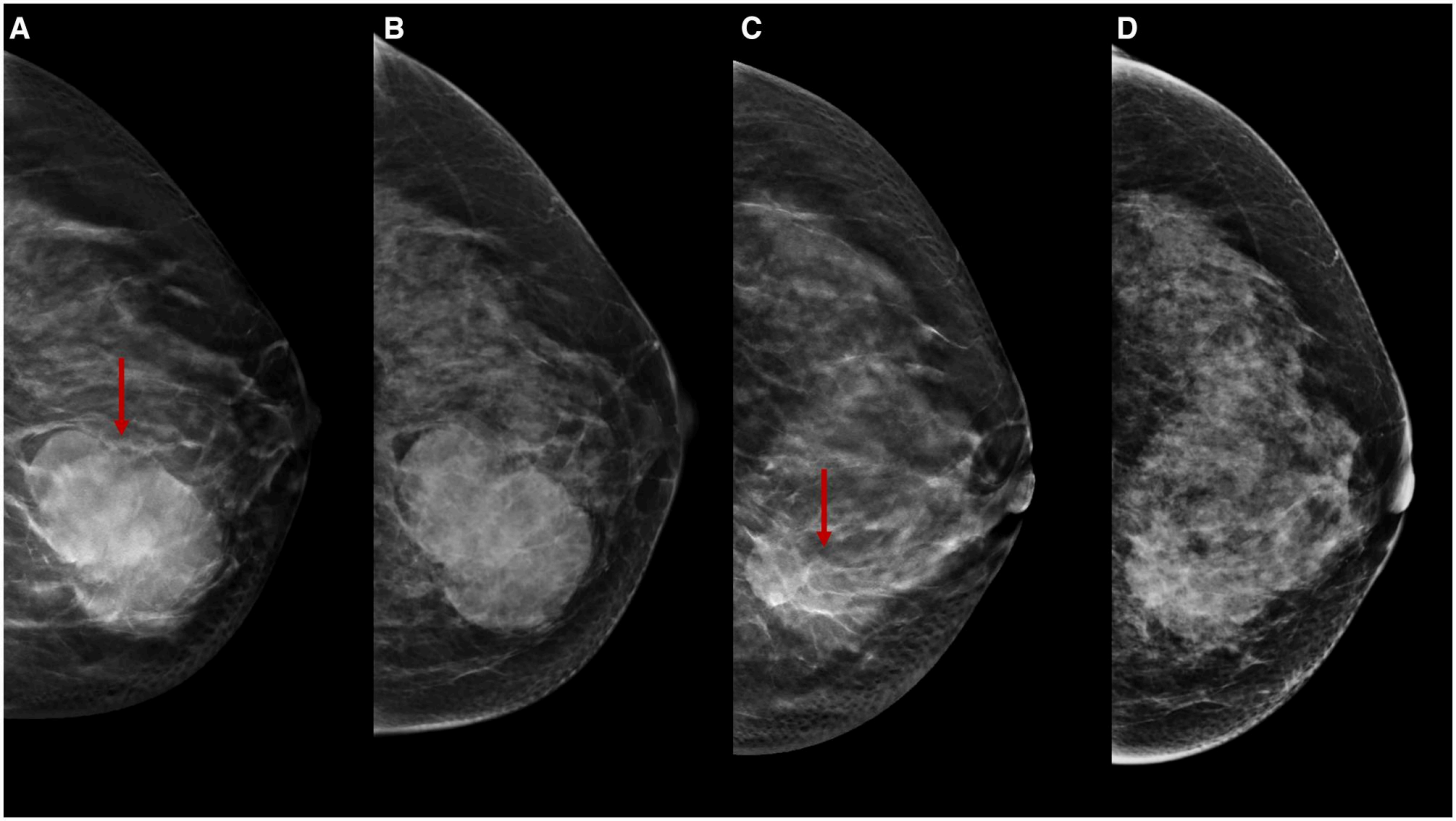
A 49-year-old woman underwent NT (four cycles of epirubicin-cyclophosphamide followed by weekly paclitaxel for 12 weeks) for an invasive ductal carcinoma cT2cN0, G2 (ER 5%, PgR 0%, Ki-67 50%, HER2 0). Cranio-caudal DBT before (A) and following (C) NT show a dimensional and density reduction of a lobulated lesion in the lower half of the left breast (arrows), an indication of treatment response, which is partially obscured by fibroglandular tissue density on DM (B, D).

On DM, breast tumours typically manifest as medium to high-density masses compared to the surrounding glandular parenchyma. Rarely (although not impossible) BC shows a lower density than normal parenchyma.^45^ Following NT, while maintaining a relatively stable size, the lesion may undergo variations in density (not always perceptible, especially in dense breasts) as an indication of possible treatment response. These changes can be attributed to the liponecrosis phenomena, a pathological process involving saponification of local fat following arterial wall damage. Liponecrosis often presents as radiolucent oval or round areas resembling luminous “bubbles” within the breast parenchyma (Figure 2).^46^^,^^47^ The use of DBT reduces tissue overlap and facilitates discrimination between tissue structures, lesions, and fat, allowing a better perception of the density variations of the lesion itself (Figure 3).

### Fibrosis

Breast stromal fibrosis is a benign histopathologic condition characterised by hypocellular fibrous tissue proliferation, leading to the obliteration of mammary acini and ducts.^48^^,^^49^ This results in the formation of localized areas of fibrous tissue with hypoplastic mammary ducts and lobules associated.^50^ After NT, tumour response may induce the formation of fibrotic regions, particularly with anthracycline-based protocols.^51^

**Figure 4.**
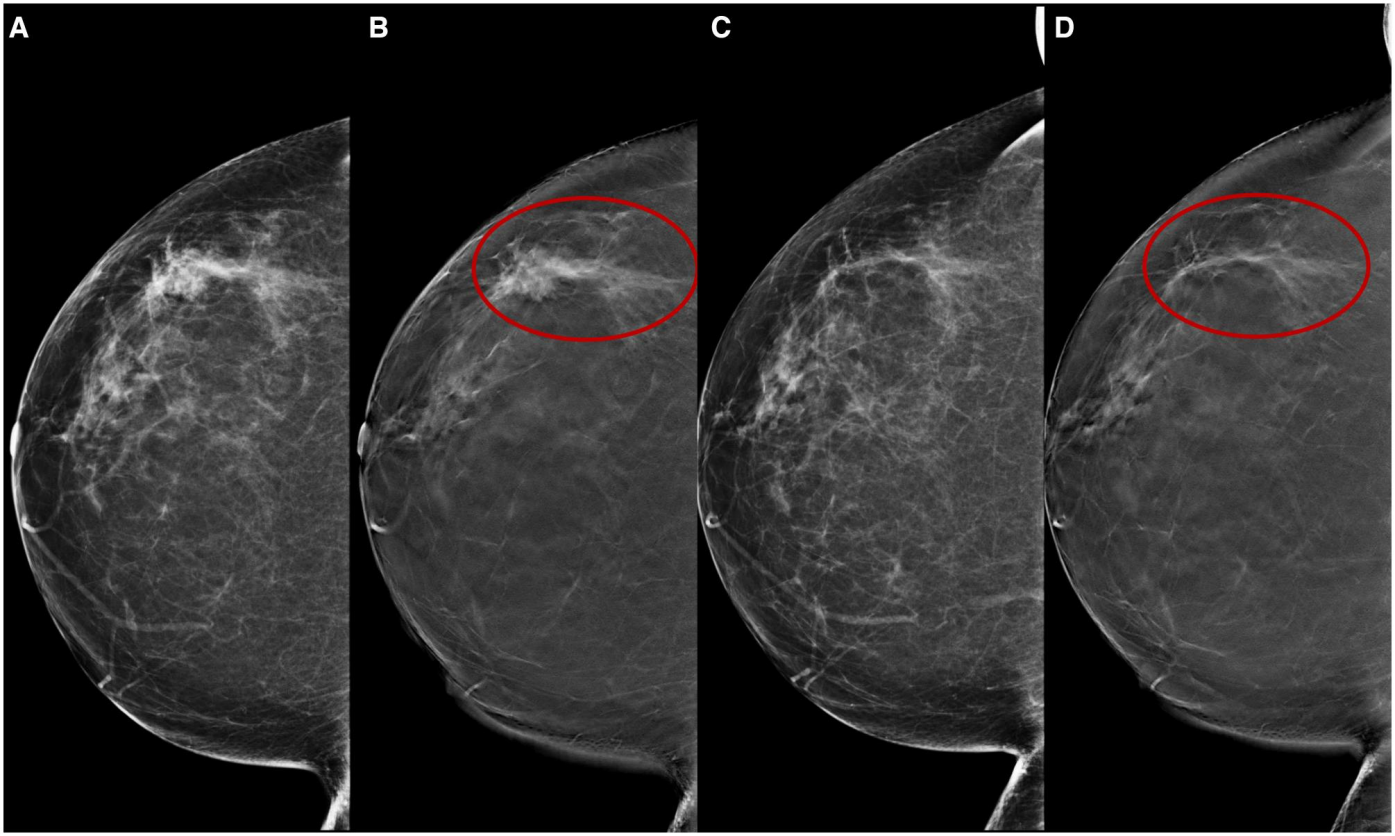
A 66-year-old woman underwent NT (four cycles of epirubicin-cyclophosphamide followed by weekly trastuzumab + paclitaxel for 12 weeks) for an invasive ductal carcinoma cT1cN0, G3 (ER 95%, PgR 0%, Ki-67 36%, HER2 3+). Cranio-caudal DBT before (B) and following (D) NT better show a residual fibrotic streak (rims) in the outer quadrants of the right breast which is not clearly seen on DM (A, B). A significant advantage of DBT is the better detection of planar lesions, such as breast structural distortions.

The imaging features of fibrosis can vary widely, although it often presents an architectural distortion with radiopaque streaks to the previous lesion site, mimicking persistent tumour disease. Fibrotic streaks typically show a decrease in density and/or size over time or may remain stable, while an increase in size or density raises suspicion of persistent disease.^46^^,^^47^ An essential benefit of DBT is the better visualisation of planar lesions, such as architectural distortions, which may also reveal fibrotic changes. Literature data confirm the enhanced sensitivity of DBT compared to DM in detecting these lesions in both fatty and dense breasts^24–26^ (Figure 4).

### DM tumour characteristics

**Figure 5.**
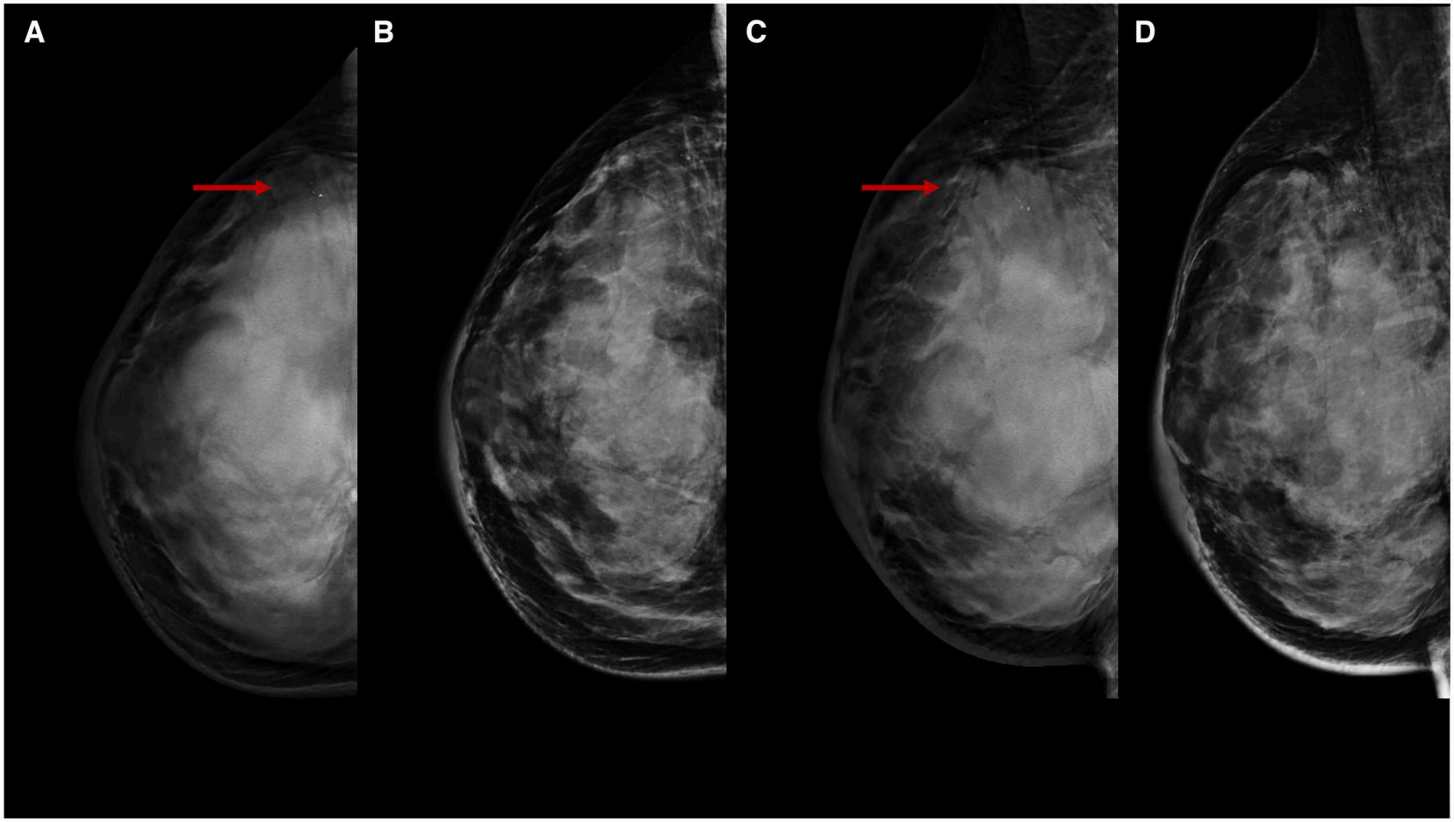
A 40-year-old woman underwent NT (four cycles of epirubicin-cyclophosphamide followed by weekly paclitaxel for 12 weeks) for an invasive ductal carcinoma cT1cN0, G3 (ER 0%, PgR 0%, Ki-67 85%, HER2 0). Cranio-caudal (A) and medio-lateral-oblique (C) DBT of the right breast before NT show an irregular opacity lesion with punctate microcalcifications in the upper outer quadrant of the right breast (arrows), which is not clearly detectable on DM (B, D). DBT allows a better assessment of lesions with irregular margins.

The DM accuracy in predicting residual tumour size after NT appears to be influenced by the initial DM characteristics of the lesion, especially by tumour margins. According to Huber et al,^52^ there is a high level of DM accuracy in the assessment of post-NT residual lesion size (77%) for tumours represented by masses with well-defined margins (>50% visibility on DM at baseline). Conversely, tumours characterised by masses with ill-defined margins exhibit a low correlation between DM and histopathological evaluation. Several studies agree on a higher correlation between size measured by DBT and definitive histology than DM.^22^^,^^23^ Mainly, DBT shows a high correlation with definitive histology in masses with spiculated margins, measuring only the core of the lesion rather than the maximum extension, including spicules^22^^,^^23^^,^^27^^,^^28^ (Figure 5).

### Presence of microcalcifications

Assessing the extent of microcalcifications on DM after NT does not always correlate with residual tumour burden and is often not representative of viable tumour tissue.^53^^,^^54^ A retrospective analysis involving 494 BC patients (106 of whom had microcalcifications) showed an association between microcalcifications identified by DM after NT with BC and benign changes in 49% and 41% of cases, respectively, and a better correlation between oestrogen receptor-positive (ER+) tumours and residual malignant microcalcifications.^53^

**Figure 6.**
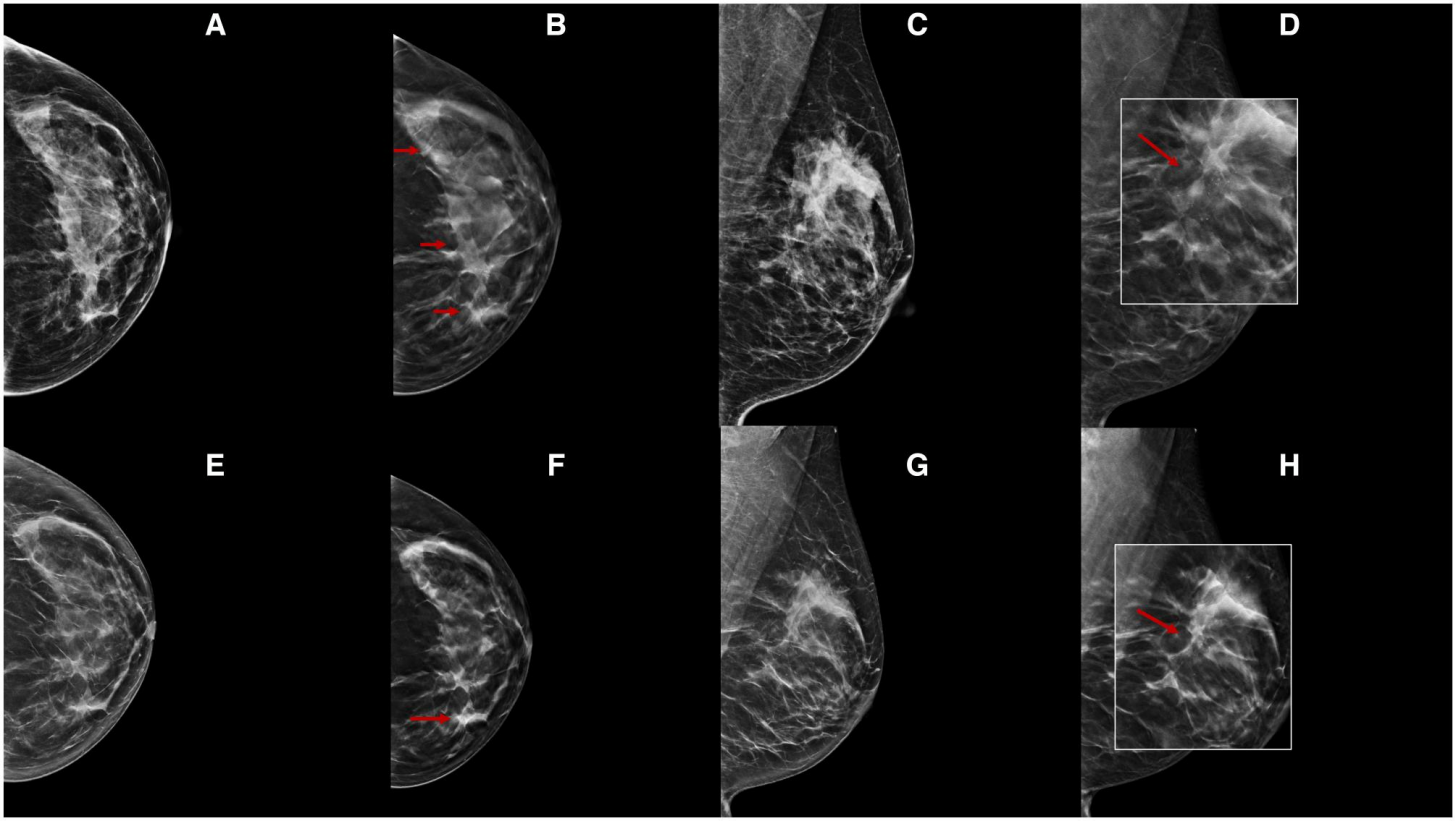
A 54-year-old woman underwent NT (four cycles of epirubicin-cyclophosphamide followed by weekly trastuzumab + paclitaxel for 12 weeks) for an invasive ductal carcinoma cT1cN0, G2 (ER 20%, PgR 1%, Ki-67 35%, HER2 3+). Cranio-caudal and medio-lateral-oblique DBT before (B, D) and following (F, H) NT better highlight many parenchymal distortion areas with spot-like microcalcifications inside in the upper quadrants of the left breast (arrows), which is not clearly seen on DM (A, C, E, G). DBT can reduce tissue overlap and the masking effect of tissue density by better evaluating residual microcalcifications.

**Figure 7.**
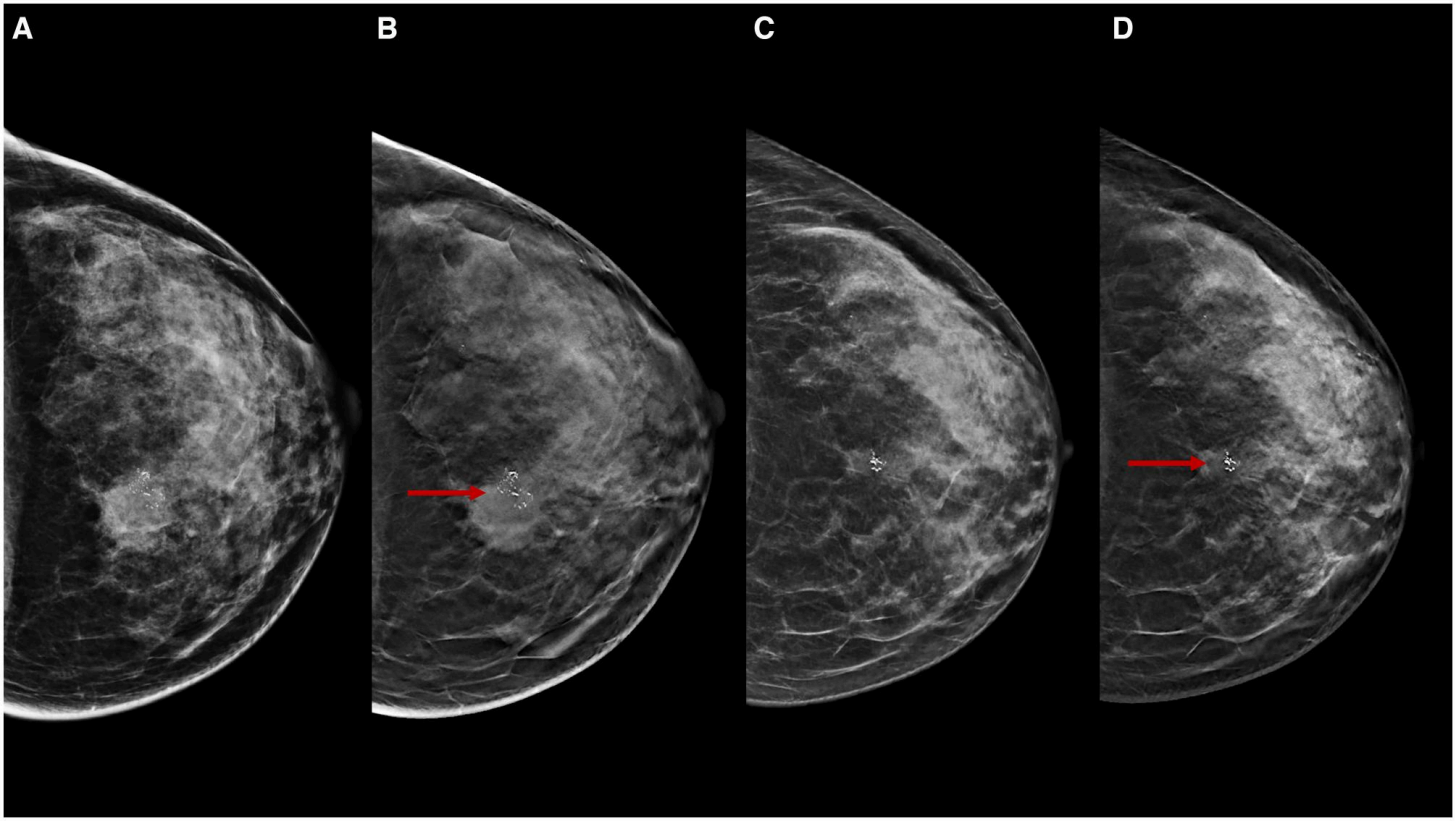
A 47-year-old woman underwent NT (four cycles of epirubicin-cyclophosphamide followed by weekly paclitaxel for 12 weeks) for an invasive ductal carcinoma cT3cN0, G3 (ER 0%, PgR 0%, Ki-67 70%, HER2 2+, FISH−). Cranio-caudal DBT before (B) and following (D) NT show an irregular lesion with pleomorphic microcalcifications associated in the upper inner quadrant of the left breast (arrows), which is better seen on DBT compared to DM (A, C). DBT facilitates discrimination between lesion opacity and calcification, emphasising their changes.

A controversial issue is the DBT's ability to detect and characterise microcalcifications. Several studies compared DBT with DM in this context, demonstrating comparable sensitivities.^55–57^ However, some calcifications may show greater conspicuity at DBT, thus improving visualisation and accurate interpretation. The almost 3D nature of DBT simplifies the identification of calcifications within the skin and facilitates the recognition of parallel alignment vascular calcifications or associated tubular vessels.^58^ Furthermore, DBT may offer advantages in depicting the distances between the clusters of calcifications and their distribution within the breast, particularly in patients with multifocal lesions, facilitating a better understanding of the disease extension for radiologists and surgeons^58^ (Figures 6 and 7). However, a crucial aspect to consider is the variability in calcification conspicuity resulting from the computational reconstruction process, potentially leading to false-positive or false-negative diagnoses. Due to synthetic DBT, an overall enhancement in calcification conspicuity has been noted compared to DM alone,^55^^,^^59^ potentially aiding in accurate image interpretation.

### Fragmentation

Following NT, several changes occur in the tumour bed, including necrosis, fibrosis, and inflammatory reactions.^60^^,^^61^ Depending on tumour size and cellularity, these alterations may exhibit different characteristics, resulting in distinct patterns of tumour shrinkage on post-treatment MRI,^57^ classified into three groups^62^^,^^63^: concentric shrinkage (CS), >3 mm without surrounding lesions; nodular shrinkage with residual multinodular lesions (NS), and mixed shrinkage (MS). Studies demonstrated a significant correlation between HER2+ tumours and CS after NT, triple negative (TN) and NS, and Luminal A cancers and MS.^63–67^

**Figure 8.**
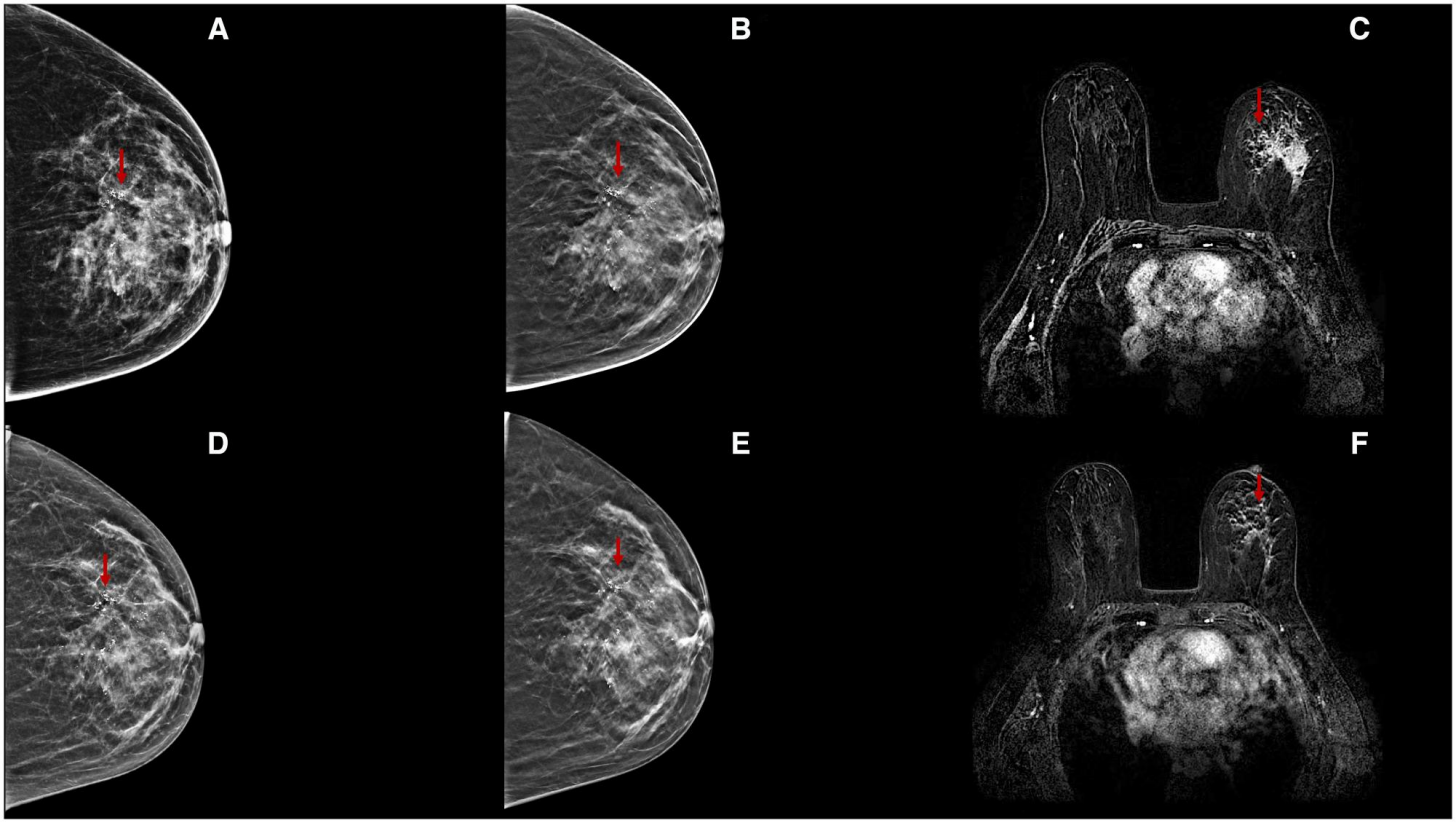
A 44-year-old woman underwent NT (four cycles of epirubicin-cyclophosphamide followed by weekly trastuzumab + paclitaxel for 12 weeks) for an invasive ductal carcinoma cT2cN0, G3 (ER 40%, PgR 0%, Ki-67 25%, HER2 3+). Cranio-caudal DBT before (B) and following (E) NT better highlight an extensive area of a pleomorphic microcalcification with increased parenchymal density in the upper half of the left breast (arrows), compared to DM (A, D). At the end of therapy, DBT allows a better assessment of the areas of radiolucency for concentric shrinkage, confirmed on MRI subtraction image (F).

DBT could potentially address the limitations of response evaluation with DM alone following NT-induced fragmentation changes, as it offers an objective assessment of the lesion overview (Figure 8).

### Molecular profiles and response patterns

BCs were classified into four molecular subtypes using oestrogen and/or progesterone receptor and HER2 status, as determined by immunohistochemistry or fluorescence *in situ* hybridisation and Ki67^68^^,^^69^: Luminal A, Luminal B, TN, and HER2+. Kim et al illustrated a significant disparity in MRI-based response patterns during and after NT between pathological responders and non-responders, proposing six classes^62^: type 0 (complete radiologic response), type 1 (CS), type 2 (NS), type 3 (diffuse contrast enhancement in whole quadrants), type 4 (stable disease, ie, no response, shrinkage <3 mm, or increase <3 mm), type 5 (progressive disease, ie, increase in tumour size >3 mm or new lesions).

A remarkable association between HER2+ tumours and CS, commonly presenting residual microcalcifications with a good or complete response rate after NT, was observed, as well as between TN and NS with a partial response rate after NT. In contrast, Luminal A cancers are often associated with MS and generally show limited or no response after therapy.^63–67^

**Figure 9.**
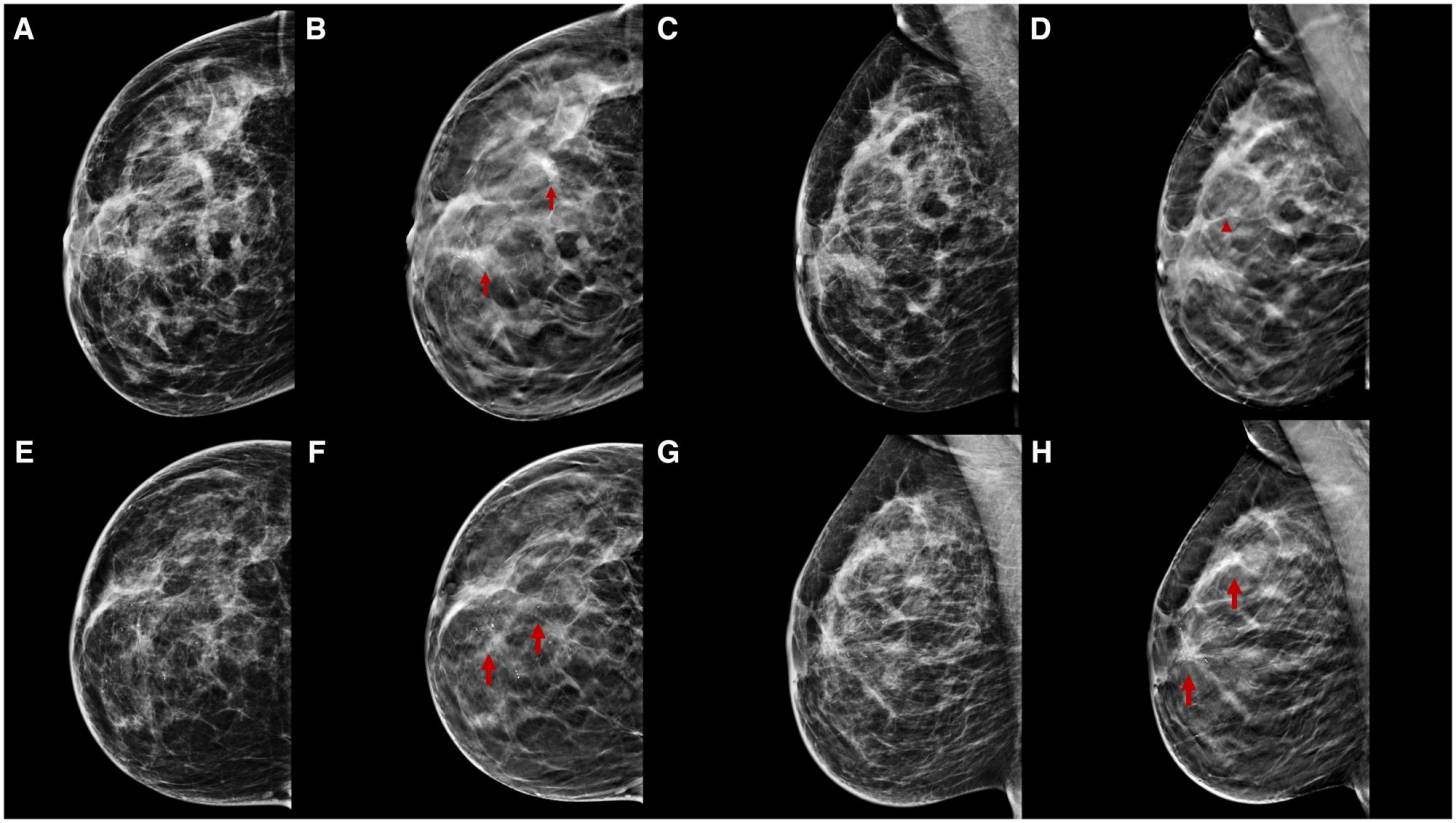
A 46-year-old woman underwent NT (four cycles of epirubicin-cyclophosphamide followed by weekly trastuzumab + paclitaxel for 12 weeks) for an invasive ductal carcinoma cT3cN0, G3 (ER 0%, PgR 0%, Ki-67 24%, HER2 3+). Cranio-caudal and medio-lateral-oblique DBT before NT (B, D) better highlight many parenchymal distortions with pleomorphic microcalcifications associated (arrows) in the upper outer breast quadrant of the right breast compared to DM (A, C). After NT, mild structural distortion with residual microcalcifications is observed on cranio-caudal and medio-lateral-oblique DBT (F, H). Histological evaluation after skin mastectomy confirms a ductal intraepithelial neoplasia (DIN 2) in fibrosis area diagnosis (ypTisN0), with a pathological complete response.

**Figure 10.**
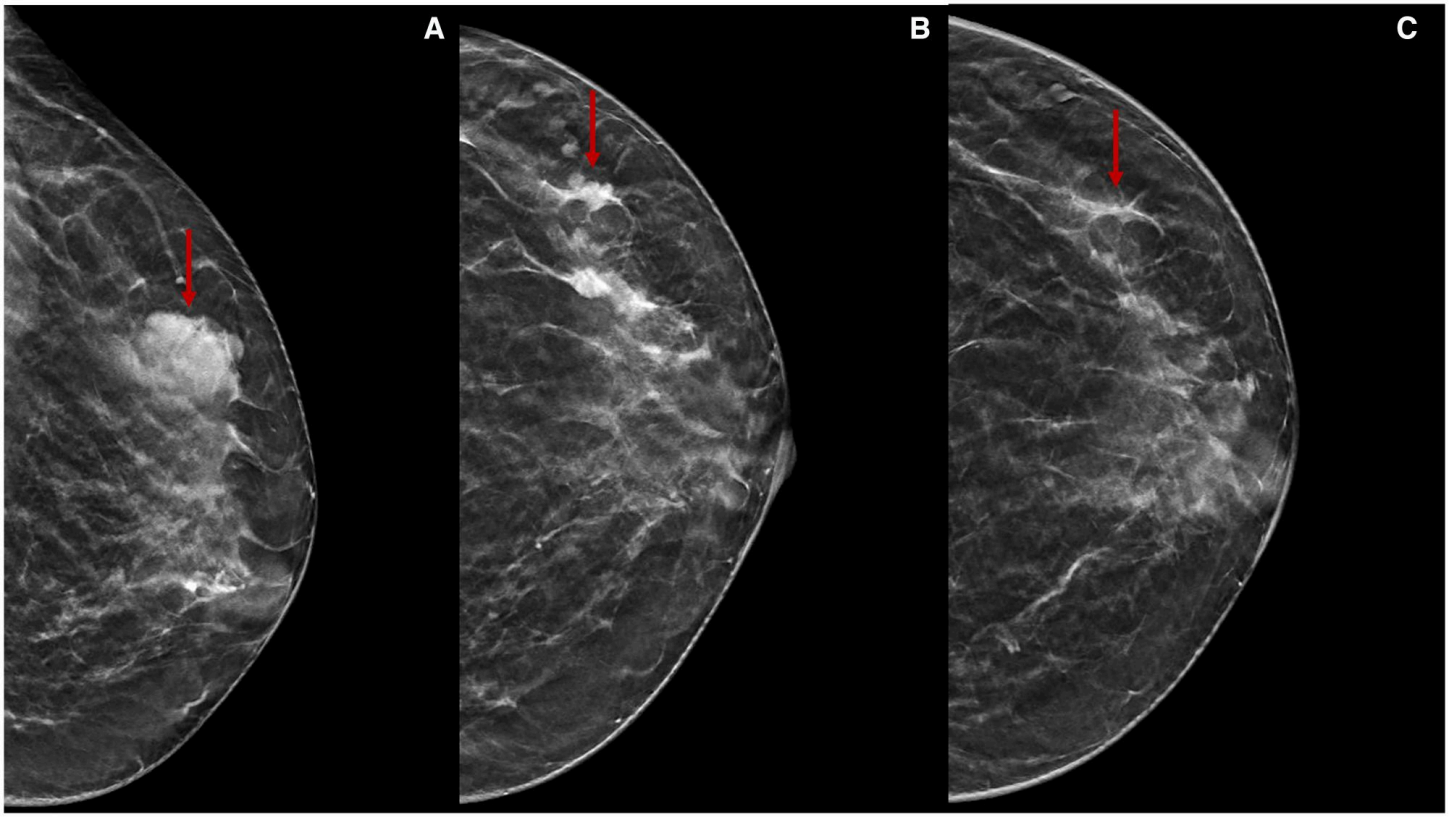
A 38-year-old woman underwent NT (four cycles of epirubicin-cyclophosphamide followed by weekly paclitaxel for 12 weeks) for an invasive ductal carcinoma cT1cN0, G3 (ER 0%, PgR 0%, Ki-67 70%, HER2 0). Cranio-caudal DBT before (A), during (B), and after (C) NT show an irregular lesion (arrows) in the upper outer quadrant of the left breast. After NT, the lesion is not observed. Histological evaluation after conservative surgery confirms a fibrosis area and residual tumour inside diagnosis (ypT1aN0), with pathological partial response.

Although MRI is the gold technique for residual disease post-NT evaluation, it can both overestimate or underestimate residual tumour size^70^; integrating DBT into conventional DM could be a promising alternative, particularly for intermediate assessments of response to therapy (Figures 9 and 10).

## Conclusions

NT plays a pivotal role in the management of BC, aiming to reduce tumour burden, optimise surgical outcomes, and provide predictive insights into treatment response. In this context, adequate radiological assessment during and after NT is essential for guiding treatment decisions and surgical planning, with the MRI considered the gold standard technique for determining disease extent and response to treatment and CEM as a valid alternative. Although DM and US are reliable tools for determining tumour size at diagnosis, secondary changes to NT can be complex to assess using conventional imaging. DBT should be considered a valid addition to DM in this situation based on its well-known potential advantages. This technology's ability to reduce tissue overlap, facilitate discrimination between normal and abnormal structures, and provide 3D perspectives contributes to improved sensitivity, showing good correlation and agreement with pathology for residual tumours after NT. Additional investigations are warranted to establish its effective role in routine clinical practice. However, the integration of DBT with conventional DM shows promise in delivering cost-effective and readily accessible imaging options, particularly beneficial for patients with contraindications to MRI or residing in areas with limited access to contrast-enhanced imaging techniques facilities.
